# Thermodynamic optimization subsumed in stability phenomena

**DOI:** 10.1038/s41598-020-71130-7

**Published:** 2020-08-31

**Authors:** J. Gonzalez-Ayala, A. Medina, J. M. M. Roco, A. Calvo Hernández

**Affiliations:** 1 Instituto Universitario de Física Fundamental y Matemáticas (IUFFyM), Universidad de Salamanca, 37008 Salamanca, Spain; 2 Departamento de Física Aplicada, Facultad de Ciencias, 37008 Salamanca, Spain

**Keywords:** Nonlinear phenomena, Statistical physics, Thermodynamics

## Abstract

In the present paper the possibility of an energetic self-optimization as a consequence of thermodynamic stability is addressed. This feature is analyzed in a low dissipation refrigerator working in an optimized trade-off regime (the so-called Omega function). The relaxation after a perturbation around the stable point indicates that stability is linked to trajectories in which the thermodynamic performance is improved. Furthermore, a limited control over the system is analyzed through consecutive external random perturbations. The statistics over many cycles corroborates the preference for a better thermodynamic performance. Endoreversible and irreversible behaviors play a relevant role in the relaxation trajectories (as well as in the statistical performance of many cycles experiencing random perturbations). A multi-objective optimization reveals that the well-known endoreversible limit works as an attractor of the system evolution coinciding with the Pareto front, which represents the best energetic compromise among efficiency, entropy generation, cooling power, input power and the Omega function. Meanwhile, near the stable state, performance and stability are dominated by an irreversible behavior.

## Introduction

Thermodynamic optimization is a response to requirements on energy production and its efficient use. Ranging from technological devices to the most basic biological energetic mechanisms the search for a compromise between producing fast with reduced losses in the process have settled the road in the energy conversion race. From an anthropological point of view, the need to satisfy an energetic demand with the minimum impact to the environment is key. In this context, operation regimes for energy converters have been studied historically passing from maximum efficiency paradigm to maximum power and compromise criteria in between, but always relying on our capacity to provide stable and controlled operation environments. On the other hand, in the biological realm, evolutionary and adaptation mechanisms, with clear energetic implications, try to guarantee survival under a wide variety of needs and variable circumstances. Although we may be far from synthesizing these mechanisms to simple ones, we have reasons enough to link a wide variety of phenomena with issues arising from energy constraints.

Due to its broad conceptual scope and applicability, thermodynamics has been widely applied in all areas of science through key concepts such as thermal equilibrium, heat fluxes or entropy generation. However, a formalization for the study of non-equilibrium processes is still under development and the proposal, study and optimization of non-equilibrium heat devices are issues in constant evolution. Especially relevant is the energetic optimization of heat devices, either heat engines (HE) or refrigerators (RE). And key aspects in the optimization of energy converters related with the second law of thermodynamics are the entropy generation and the thermal efficiency/power output for HE’s or coefficient of performance/cooling rate for RE’s^1–5^.

Significant efforts have been devoted to study the linkage between different heat device models by means of their performance at certain operation regimes with minimum model-dependent characteristics. Most of them have been focused in the context of HE, nonetheless, a unified outlook of general properties for any kind of energy converters is highly desirable.

Beyond the maximum power or maximum cooling rate operation regimes, the practical requirement of a better and sustainable use of energy have resulted in the proposal of trade-off figures of merit, involving compromises among maximum power/cooling rate, minimum entropy generation and maximum efficiency/coefficient of performance (COP), including exergetic and thermo-economic merit functions^6–8^.

In particular, the so-called low-dissipation (LD) model allows for a quite straightforward thermodynamic interpretation in the analysis of unified heat devices^9–15^. This model is suitable for exploring general irreversible behaviors not linked with particularities of the heat transfer mechanisms. It could be understood as a first irreversible deviation from a Carnot HE (RE) and displays the capability of obtaining upper and lower bounds for the efficiency (COP) for several operation regimes without specifying heat transfer mechanisms, but focusing on the symmetries in the dissipation and in the contact times with the hot and cold reservoirs^13,14^, and optimization under fixed cooling power are being subject of research due to its practical relevance^16,17^. Its wide applicability allows its mapping onto a variety of irreversible models, such as the minimally nonlinear irreversible heat engine, a heat engine with weighted thermal fluxes, the endoreversible/irreversible Carnot-like heat engine and in stochastic heat engines^2,12,18–24^ with special emphasis in small-scale refrigerators^25–33^. Experimental data have validated this model, as it is the case of an optical trap performing a micrometric Stirling heat engine^34^ and a Brownian Carnot cycle^35^ as well as for macroscopic setups^36^.

Despite the advances in optimization, there is a specific unsolved issue: the systematic treatment of the influence of lack of control^37^. There are open questions regarding the role of constancy (fluctuations in the energetic output records)^38–40^, which could be ultimately related to power fluctuations with large efficiencies^38,39^ or the enhancement of energy converters performance due to fluctuations from small scale and quantum nature as recently proposed^41–43^. Major differences have been recently reported for quasistatic and steady state HE models operating close to the reversible efficiency^39^. Issues such as the Carnot efficiency at finite power and efficiency at maximum power have been widely analyzed to account for control of parameters and engine layouts, though key differences have been reported depending on time constraints among other factors^38,39^, hinting for a relation between reversibility and the amplitude of fluctuations.

A complementary study to the optimization of operation regimes is the analysis of their operation regime stability, that is, if operation variables have fixed values, they remain close to those values even when external stressors generate small perturbations on them, generating a time-periodic steady-state. In general, energetic landscapes are responsible for stability criteria such as it occurs in first order phase transition, partially responsible for structural composition of matter. In particular, regarding operation design, in every natural energy conversion mechanism the problem of variability of the device surroundings somehow has been solved, even under large variation on the operation conditions, such is the case of photosynthesis, to name one, with a large variability in solar radiance and temperature in time. Thus, stability must be involved somehow with operability, even more if we consider evolution as stochastic in nature.

Recently, it has been shown that a HE in an stationary state undergoing small perturbations of the energy exchanges with the surroundings obeys dynamical equations of the optimization time-variables. In that case, trajectories towards stationary state are not arbitrary and exhibit an optimization mechanism for the most relevant thermodynamic functions^44–46^. In view of this analysis, there is a promising way to incorporate stability as a new ingredient in the optimization of heat devices with new features appealing for a better understanding of a thermodynamic self-improvement stemming from stability. In this way, trade-off based figures of merit, beyond practical design and economical reasons, would arise as natural responses, with evolutionary mechanisms and adaptability to the environment^47–51^. In the same way, mechanisms stemming from thermodynamics to induce an operation state have been explored for stochastic and deterministic models^52,53^.

This connection has been previously addressed for HE’s^45,46^, the aim of this paper is to extend that study for the by far less known cooling systems, reinforcing the vision of a general behavior of HE’s and RE’s. The first goal of this paper will be to present a detailed study on fluctuations of the main energetic properties (as COP, entropy generation, cooling power, and work input) for a LD-RE around a trade-off steady state and to analyze how these magnitudes evolve around the stable point. To achieve this objective two possible dynamics for the perturbation of the system around the stable point are considered introducing two different sets of control parameters and two different stability configurations (a stable point inside a basin of attraction and an isolated stable point). The second goal is to deepen in the analysis of limited control along the cyclic process by introducing consecutive random perturbations during one cycle time and through many cycles. The evolution of the generated stochastic trajectories and their statistical behavior points out to the existence of a well-defined trend driven by the energetic improvement of the system.

## The model and the $\Omega$ regime

The LD model is a first-order irreversible deviation from a Carnot cycle, where irreversibilities are introduced only in the coupling between the working fluid and the hot and cold reservoirs at temperatures $T_c$ and $T_h$, respectively^9^. The adiabatic processes are considered as instantaneous. The time dependent character of the model is introduced by means of the so-called irreversible coefficients $\Sigma _c$ and $\Sigma _h$ for each isothermal process, divided by the time these processes last, $t_c$ and $t_h$, respectively. The fact that the baseline cycle is the Carnot one (whose efficiency does not depend on the working substance) when extending the reversible cycle to the irreversible framework allows for a structure independent description, where the specific properties of the working fluid are not relevant, and one can focus on the operation variables to optimize.

The input and output heats for a refrigerator are $Q_c=T_c\Delta S\left[ 1-\Sigma _c/(\Delta S\,t_c)\right]$ and $Q_h=T_h\Delta S\left[ -1-\Sigma _h/(\Delta S\,t_h)\right]$, where $\Delta S$ is the entropy change at the cold isotherm of the baseline reversible Carnot cycle. These expressions allow to decompose the heat exchange in a reversible component, $T\Delta S$, and an additional irreversible contribution proportional to the inverse of time. The mentioned irreversible coefficients $\Sigma _c$ and $\Sigma _h$ depend on the internal dynamics of the working fluid and thus, they are attached to intrinsic properties that for the purposes of optimization of the operation regime can be considered as constant. The total entropy change in the thermodynamic universe is given by $\Delta S_{tot}=\Sigma _c/t_c+\Sigma _h/t_h$, so that $\{t_c,t_h\}\rightarrow \infty$ (or $\{\Sigma _c,\Sigma _h\}\rightarrow 0$) the reversible performance is recovered. The main energetic quantities are the refrigerator coefficient of performance, COP $\varepsilon =-Q_c/(Q_h+Q_c)$, the cooling power, $R=Q_c/(t_c+t_h)$ and the power input $P_{in}=W/(t_c+t_h)=-(Q_h+Q_c)/(t_c+t_h)$.

The $\Omega$ function was proposed for a generic heat device as a trade-off figure of merit between the maximum useful energy and the unavoidable useful energy losses for a given energy input. For a RE, it is defined as the compromise between maximum cooling rate, $R=Q_c/t$, and lost cooling rate for a fixed power input, $P_{in}$. Then, the $\Omega$ function is^14^

1 $$\begin{aligned} \Omega =\left( 2\varepsilon -\varepsilon _C\right) P_{in}=2 R-\varepsilon _C P_{in}. \end{aligned}$$

where $\varepsilon _C=\tau /(1-\tau )$ is the COP of the Carnot cycle and $\tau \equiv T_c/T_h$. Upper and lower bounds are obtained in the limits $\Sigma _{c}\rightarrow 0$ and $\Sigma _{h}\rightarrow 0$, respectively

2 $$\begin{aligned} \varepsilon ^{M\Omega }_-=\frac{2}{3}\varepsilon _C\le \varepsilon ^{M\Omega } \le \frac{3+2\varepsilon _C}{4+3\varepsilon _C}\varepsilon _C=\varepsilon ^{M\Omega }_+. \end{aligned}$$

In the symmetric case $\Sigma _c=\Sigma _h$^14^,

3 $$\begin{aligned} \varepsilon ^{M\Omega }_{sym}=\frac{\varepsilon _C}{\sqrt{(1+\varepsilon _C) (2+\varepsilon _C)}-\varepsilon _C}. \end{aligned}$$

The optimization of this function can be achieved equivalently using two different sets of time variables. In one case the total time and one partial contact time are used, allowing to reproduce behaviors typical from endoreversible and irreversible devices. In the other case the two partial contact times are considered. Different but complementary dynamical equations for the stability of this operation regime are obtained in each case. In the next section the first option is analyzed.

## On time constraints, multi-objective optimization and stability

A fruitful representation of the system is achieved by using the operation total time and one partial contact time. This choice of optimization variables allows to recover endoreversible and irreversible behaviors which are tightly related with the multi-objective optimization of the RE, as will be shown later and it happens in the case of LD-HE’s^45,46^. With this purpose the dimensionless variables, ${\widetilde{\Sigma }}_c\equiv \Sigma _c/\Sigma _T$, $\alpha \equiv t_c/(t_c+t_h)$, ${\widetilde{t}}\equiv \Delta S\,(t_c+t_h)/\Sigma _T$, with $\Sigma _T=\Sigma _h+\Sigma _c$ that account for the system size are considered. From these definitions it is possible to work with dimensionless input and output (cooling power) heat fluxes^11^,

4 $$\begin{aligned} {\dot{{\widetilde{Q}}}}_c\equiv \frac{Q_c}{{\widetilde{t}}\;T_h\,\Delta S}={\widetilde{R}}=\left( 1-\frac{{\widetilde{\Sigma }}_c}{\alpha {\widetilde{t}}}\right) \frac{\tau }{{\widetilde{t}}}; {\dot{{\widetilde{Q}}}}_h\equiv \frac{Q_h}{{\widetilde{t}}\;T_h\,\Delta S}=-\left( 1+\frac{1-{\widetilde{\Sigma }}_c}{(1-\alpha ){\widetilde{t}}}\right) \frac{1}{{\widetilde{t}}}. \end{aligned}$$

A total entropy production, scaled with the size of the baseline Carnot cycle, can be obtained ${\widetilde{\sigma }}\equiv \Delta S_{tot}/({{\widetilde{t}}}\,\Delta S)= [{{\widetilde{\Sigma }}}_c/\alpha +(1-{{\widetilde{\Sigma }}}_c)/(1-\alpha )]/{\widetilde{t}}^2$. And from Eq. (4), the input power, ${\widetilde{P}}_{in}=-{\dot{{\widetilde{Q}}}}_c-{\dot{{\widetilde{Q}}}}_h$; the COP, $\varepsilon ={{\widetilde{R}}}/{{\widetilde{P}}}_{in}$ and ${{\widetilde{\Omega }}}\equiv (2\varepsilon -\varepsilon _C){\widetilde{P}}_{in}$ can be calculated.

The optimization variables are $\alpha$ and ${{\widetilde{t}}}$, meanwhile $\tau$ and ${{\widetilde{\Sigma }}}_c$ are fixed parameters, referring to external operation conditions and intrinsic material properties, respectively. The values of $\alpha$ and ${{\widetilde{t}}}$ that maximize $\Omega$ are:

5 $$\begin{aligned} \alpha ^{M\Omega }=\frac{1}{1+\sqrt{\frac{1-{\widetilde{\Sigma }}_c}{\left( 2-\tau \right) {\widetilde{\Sigma }}_c}}}; {\widetilde{t}}^{M\Omega }=\frac{2}{1-\tau }\left( \sqrt{\left( 2-\tau \right) {\widetilde{\Sigma }}_c}+\sqrt{1-{\widetilde{\Sigma }}_c}\right) ^2 . \end{aligned}$$

A common feature in a variety of irreversible models is the appearance of loop-like power-efficiency parametric curves in heat engines and $\chi$–$\varepsilon$ curves for refrigerators. This has been considered as a characteristic signature of irreversibility. However, the so-called endoreversible model^54^ consisting of a reversible Carnot engine irreversibly coupled to external heat reservoirs exhibits parabolic behaviors on those curves. In previous works it was shown that by fixing the value of $\alpha$ or ${{\widetilde{t}}}$ in the low dissipation model, it is possible to recover those types of behaviors. In the first case $\alpha =\alpha ^{M\Omega }$ while ${{\widetilde{t}}}$ can take values from 0 to $\infty$ (allowing to recover the reversible limit when ${{\widetilde{t}}}\rightarrow \infty$). For the latter $\widetilde{t}={{\widetilde{t}}}^{M\Omega }$ and $\alpha$ takes values from 0 to 1 (this fixes the irreversibility of the system, since the reversible limit cannot be achieved). These two behaviors are discussed in detail in^13^, as part of a unified phenomenology for HE’s and RE’s and will become relevant in the present analysis of the stability-optimization relation. Some insights on the role of irreversible and endoreversible behaviors in the stability and optimization are also discussed for HE’s in^45^.

Since the relaxation dynamics will be linked to an optimization process, a multi-objective optimization will be presented below.

### The best energetic compromise

It would be highly desirable to maximize the coefficient of performance, cooling power and simultaneously minimize entropy production and power input. However, no configurations can be found that fulfill all these requirements. It is common to search, instead, for the so-called Pareto front, which gives the best performance when one is looking to optimize simultaneously several objective functions^55^.

In this treatment two complementary outputs will be pursued: the location of the Pareto front, and second, the location of these points in the phase space (the Pareto optimal set) to compare them with the time constraints and the stability nullcline which will be discussed in in “Consecutive random perturbations” section.

The usual concept of dominance is used: in order to build the Pareto set a vector $v=(v_1,\ldots ,v_n)$ dominates another one $w=(w_1,\ldots ,w_n)$ if and only if $v_i\ge w_i\;\forall \,i\in \{1,\ldots ,n\}$ (if one in looking for a maximum, $\le$ for a minimum) and there is at least one *j* such that $v_j>w_j$. In other words, the improvement of any function will degrade some others. Here the vector is formed by the COP, $\Omega$, ${{\widetilde{P}}}_{in}$, ${{\widetilde{R}}}$ and ${{\widetilde{\sigma }}}$. The algorithm introduced here is a modification of the one introduced in^45,46^, and is as follows:

1. In the phase space ($\alpha$,${{\widetilde{t}}}$), the region of physical relevance is defined ($\varepsilon >0$ and $R>0$).
2. A random set of points in the phase space is obtained and the thermodynamic functions are evaluated (energetic space).
3. The set of non-dominated points in the energetic space is obtained, giving a provisional Pareto front.
4. From the corresponding Pareto optimal set (phase space) a convex region is defined and extended in order to cover a larger region for searching new points in the Pareto front. Details on the definition of the extended region are given below.
5. From the new region a new set of random points is proposed and a new set of non-dominated points in the energetic space is obtained.

In the present analysis, the Kullback–Leibler divergence (KLD)^56^ is calculated between the distribution of points of the *i*th and the $i-1$th iterations. The radii to extend the search region in the phase space decreases with the KLD value. When this relative entropy is very small, there is no information gain in iterating more times, then, the search for new points in the Pareto optimal set stops.

**Figure 1:**
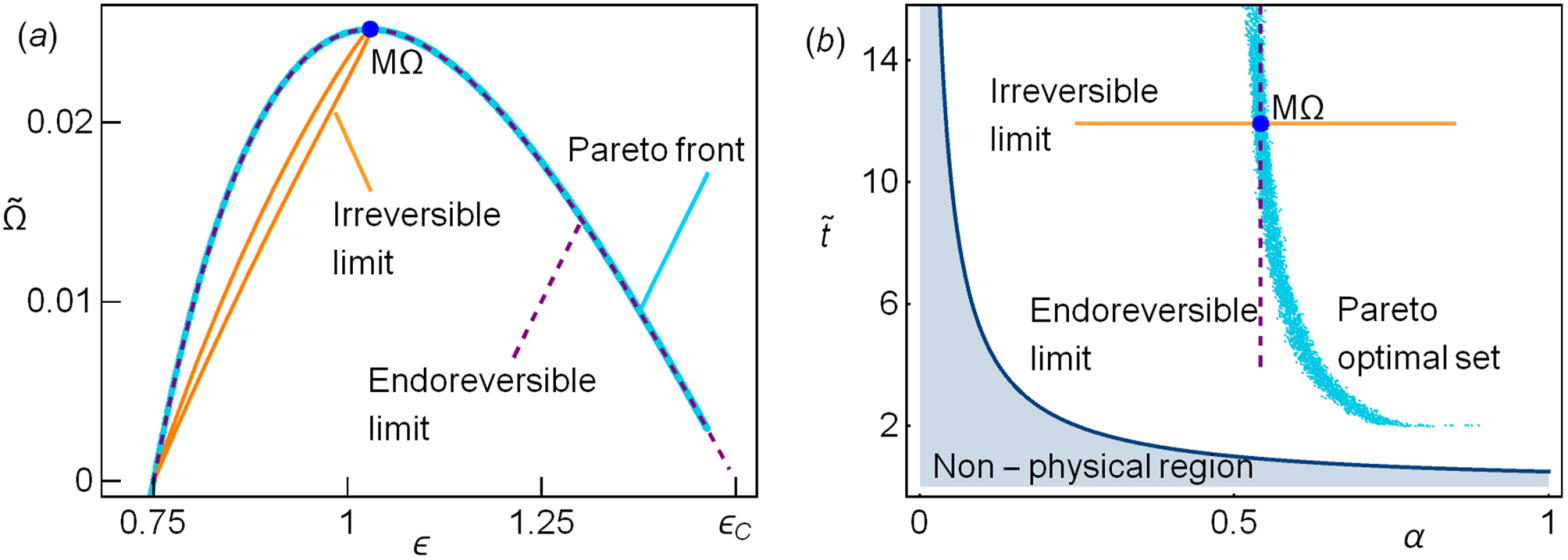
(**a**) The Pareto front (cyan points) matches the endoreversible limit (parabolic dashed line). For completeness, the irreversible limit is also depicted (loop-like curve). (**b**) The Pareto optimal set is shown (cyan points). The constraints $\alpha =\alpha ^{M\Omega }$ (horizontal line) and ${\widetilde{t}}={\widetilde{t}}^{M\Omega }$ (vertical dashed line) are shown. The representative values $\tau =3/5$ and ${{\widetilde{\Sigma }}}_c=1/2$ have been considered.

In Fig. 1a the Pareto front is shown using as objective function ${\widetilde{\sigma }}$ (minimization), $\varepsilon$, ${{\widetilde{\Omega }}}$ and ${{\widetilde{R}}}$ (maximization). The consideration of more functions, such as ${{\widetilde{P}}}_{in}$ does not contribute to obtain new points. For a LD-HE it was found that the Pareto front was remarkably close to the endoreversible limit. In this case the match is higher. Thus, this limit offers the best energetic compromise. The corresponding points in the phase space are depicted in Fig. 1b, along with the boundary of the physical region of interest (where the efficiency and the cooling power are positive).

### Stability dynamics

The problem of stability is frequently tackled through a first order equations system. By means of a linearization about the stable point, in many cases the resulting dynamics has the form $f(y)=-dy/dt\equiv -{\dot{y}}$, being *y* the dynamical variable and *t* the dynamical time. This is the kind of dynamical equation describe the stability of *RC* electrical circuit, overdamped spring, or any stable point in a potential well, *V*(*y*), near its minimum ($y^*=0$) and where $\dot{y}=-dV/dy$.

6 $$\begin{aligned} V(y)=V(0)+\left. \frac{dV}{dy}\right| _0y+\frac{1}{2}\left. \frac{d^2V}{dy^2}\right| _0 y^2+{\mathscr{O}}(y^3)\approx \frac{k}{2} y^2+V(0), \end{aligned}$$

which is the well-known approximation to a harmonic oscillator. This is the kind of dynamics appearing in colloidal particles undergoing micrometric Carnot and Stirling heat engines through optical traps, that experimentally validated the low dissipation model^34,35^.

Below, it will be shown that if the operation regime of a time-periodic heat device has a steady state (an equilibrium point specified through the operation variables), for sufficiently small perturbations, it is expected that the (probably yet unknown) dynamics of the system can be expressed as a first order system. By means of a Taylor expansion of energetic functions (in this case ${{\widetilde{Q}}}_c$ and ${{\widetilde{\Omega }}}$) around the steady state, a generic first order system can be transformed to a dynamical equation linking the operation variables and the energetic functions, allowing to effectively associate variations on the operation regime to fluctuations on the heat fluxes between the system and the heat reservoirs. In this way external perturbations can be linked to variations of the operation times (affecting energy fluxes). Notice that these perturbations come from external sources, and are not linked to the internal dynamics, already accounted by the coefficients ${{\widetilde{\Sigma }}}_c$ and ${{\widetilde{\Sigma }}}_h$.

As mentioned above, let us focus only on external perturbations on the operation regime (and for extension, to the variables that define such regime). The starting point is to assume that the system has an equilibrium point at the operation regime. With no further information regarding the specific energy transport, ${{\widetilde{t}}}$ and $\alpha$ are assumed to follow, within the first order scheme^57^, a typical (and the simplest) relation for an autonomous system in one dimension given by a dynamical equation ${\dot{Y}}=-{\mathscr{A}}\;Y$, where $Y=\left( \alpha -\alpha ^{M\Omega },{{\widetilde{t}}}-\widetilde{t}^{M\Omega }\right)$. The dynamical time, *t* has no dimensions and has a characteristic timescale that will be chosen later and ${{\mathscr{A}}\in {\mathscr{M}}_{2\times 2}}$.

The natural energy flux associated to the time variable $\alpha$ would be ${{\widetilde{Q}}}_c$ (variations of $\alpha$ will produce changes in the input heat). Meanwhile ${{\widetilde{\Omega }}}$, a global energetic function will be linked to variations on the total time ${{\widetilde{t}}}$. Analogous results are obtained if another global function is chosen, such as the so-called $\chi$ function (the equivalent to power output for heat engines^13^).

From a first order expansion of $\dot{{{\widetilde{Q}}}}_c$ and ${{\widetilde{\Omega }}}$ around the steady state ($Y=0$) one obtains

7 $$\begin{aligned} \left[ {\begin{array}{c} \dot{{{\widetilde{Q}}}}_c\left( \alpha ,{{\widetilde{t}}}\right) -\dot{\widetilde{Q}}_c\left( \alpha ^{M\Omega },{{\widetilde{t}}}^{M\Omega }\right) \\ {{\widetilde{\Omega }}}\left( \alpha ,{{\widetilde{t}}}\right) -\widetilde{\Omega }\left( \alpha ^{M\Omega },{{\widetilde{t}}}^{M\Omega }\right) \\ \end{array} }\right] \approx {\mathscr{J}}\; \left[ {\begin{array}{c} \alpha -\alpha ^{M\Omega } \\ {{\widetilde{t}}}-{{\widetilde{t}}}^{M\Omega } \\ \end{array} }\right] . \end{aligned}$$

with ${\mathscr{J}}$ the Jacobian matrix

8 $$\begin{aligned} {\mathscr{J}}=\left[ {\begin{array}{cc} \left. \frac{d\dot{{{\widetilde{Q}}}}_c\left( \alpha ,\widetilde{t}\right) }{d\alpha }\right| _{M\Omega } &{} \left. \frac{d\dot{\widetilde{Q}}_c\left( \alpha ,{{\widetilde{t}}}\right) }{d\widetilde{t}}\right| _{M\Omega } \\ \left. \frac{d{{\widetilde{\Omega }}}\left( \alpha ,\widetilde{t}\right) }{d\alpha }\right| _{M\Omega } &{} \left. \frac{d\widetilde{\Omega }\left( \alpha ,{{\widetilde{t}}}\right) }{d\widetilde{t}}\right| _{M\Omega } \\ \end{array} }\right] . \end{aligned}$$

Then, ${\dot{Y}}=-{\mathscr{A}}\;Y$ can be written as

9 $$\begin{aligned} \frac{d\alpha }{dt}= & {} C\left( \dot{{\widetilde{Q}}}_c(\alpha ^{M\Omega }, {\widetilde{t}}^{M\Omega })-\dot{{\widetilde{Q}}}_c(\alpha ,{\widetilde{t}})\right) , \end{aligned}$$

10 $$\begin{aligned} \frac{d{\widetilde{t}}}{dt}= & {} D\left( {\widetilde{\Omega }}(\alpha ^{M\Omega }, {\widetilde{t}}^{M\Omega })-{\widetilde{\Omega }}(\alpha ,{\widetilde{t}})\right) , \end{aligned}$$

where $${\mathscr{A}}=-\left[ {\begin{array}{cc} C &{} 0 \\ 0 &{} D \\ \end{array} }\right] \,{\mathscr{J}}$$; *C* and *D* are positive constants determining the response speed to perturbations from the steady state that we will refer as the restitution strength^45^. Their values may depend on multiple characteristics, but usually the system size is the most important. Because large systems are more likely to respond slowly to perturbations on the control variables than small systems, the larger the system the smaller the values of *C* and *D*. From a dynamical perspective, their inverse values set a characteristic time scale, so that large values of *C* and *D* correspond to large restitution strength and short characteristic times. In the forthcoming analyses, results are referred to this time scale.

Notice that the dynamical Eqs. (9) and (10) are not known a priori, but they are a mathematical requirement from stability relying, of course, on the assumption that the operation regime has a steady state. There are however, plausible arguments to think this is the case for several systems, since natural energy converters have displayed robustness, the capability to maintain and change operation regimes under stress in the operation conditions^62^.

In the linear approximation, the local stability of the above-given steady state is determined by the Jacobian matrix in Eq. (8) which by definition, in the M$\Omega$ regime

11 $$\begin{aligned} {\mathscr{J}}=-C \left[ \begin{array}{cc} \left. \frac{\partial \dot{{\widetilde{Q}}}_c}{\partial \alpha }\right| _{M\Omega } &{} \left. \frac{\partial \dot{{\widetilde{Q}}}_c}{\partial {\widetilde{t}}}\right| _{M\Omega } \\ 0 &{} 0 \end{array} \right] . \end{aligned}$$

The determinant of ${\mathscr{J}}$ is zero as well as one eigenvalue; the other one is given by $\lambda _1=C\left. \partial \dot{{\widetilde{Q}}}_c /\partial \alpha \right| _{M\Omega }=C\,\tau \,{{\widetilde{\Sigma }}}_c/\left( \alpha ^{M\Omega }\widetilde{t}^{M\Omega }\right) ^2$, leading to a relaxation time ($t_1=\lambda _1^{-1}$)

12 $$\begin{aligned} t_1=\frac{\left( \alpha ^{M\Omega }\widetilde{t}^{M\Omega }\right) ^2}{C\tau {{\widetilde{\Sigma }}}_{\mathrm{c}}}= \frac{4(2-\tau )\left( \sqrt{1-{{\widetilde{\Sigma }}}_c} +\sqrt{(2-\tau ){{\widetilde{\Sigma }}}_c}\right) ^2}{(1-\tau )^2\tau \;C}= \frac{2(2-\tau )}{\tau \,(1-\tau )\;C}\;{\widetilde{t}}^{M\Omega }. \end{aligned}$$

**Figure 2:**
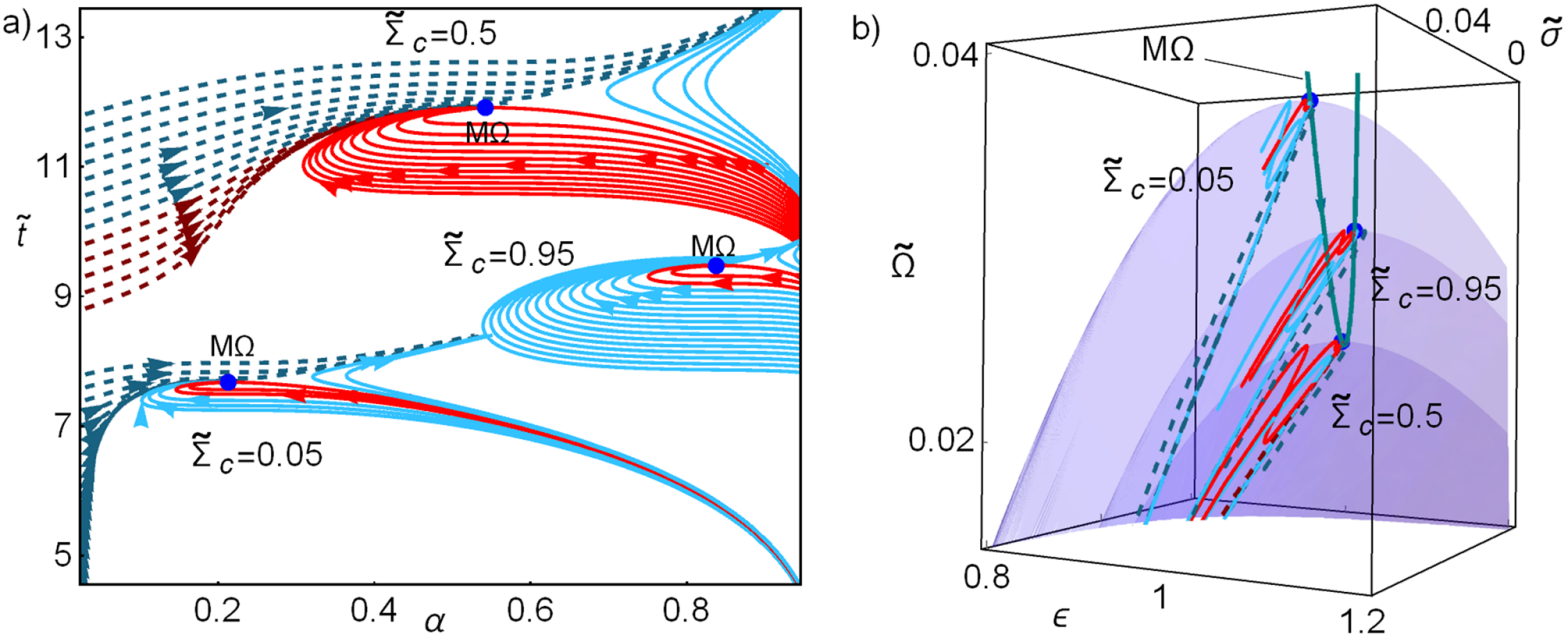
Some representative trajectories given by solving numerically Eqs. (9) and (10). The symmetry in the dissipation coefficient ${{\widetilde{\Sigma }}}_c$ affects the shape of the basin of attraction. In (**a**) three cases are depicted: ${{\widetilde{\Sigma }}}_c=\{0.05,\,0.5,\,0.95\}$, the symmetric case exhibits the largest stability basin. In (**b**) some trajectories are mapped into the energetic surface with $\tau =3/5$. The red curves indicate trajectories inside the basin of attraction; dashed (red and blue online) curves denote those trajectories with initial conditions $\alpha \rightarrow 0$ and in continuous lines (red and blue) those with initial conditions $\alpha \rightarrow 1$.

The relaxation time, $t_1$ (which refers to the variable $\alpha$), and the operation time, ${\widetilde{t}}^{M\Omega }$, are linked, being the first indication that the two phenomena are related. In the stability of a cyclic process one would require that $t_1\le {{\widetilde{t}}}^{M\Omega }$, leading to the constraint

13 $$\begin{aligned} C\ge \frac{2(2-\tau )}{\tau (1-\tau )}. \end{aligned}$$

Beyond the linear approximation presented above, the system of equations given by Eqs. (9) and (10) can be numerically solved. This dynamic, as in the case of HE’s, produce stability basins, see Fig. 2. Trajectories inside the stability basin will arrive to the stationary state, the rest will diverge to a non-desirable state of infinite operation time and null cooling power. Even when the constraint provided by Eq. (12) does not involve *D*, it affects the stability basin shape which depends additionally on $\tau$ and ${{\widetilde{\Sigma }}}_c$. Figure 2 shows the influence of the dissipation coefficient (${{\widetilde{\Sigma }}}_c$) on the basin of attraction for the complete solution obtained by solving numerically Eqs. (9) and (10) for given initial conditions/states (state after a perturbation). In Fig. 2a some representative trajectories for three dissipation symmetries, ${{\widetilde{\Sigma }}}_c=\{0.05,\,0.5,\,0.95\}$, are depicted in the phase space. All the trajectories evolve in such a way that the variable ${{\widetilde{t}}}$ does not decrease. This is intuitively understood as this variable is related to the irreversibility of the system. In Fig. 2b, some trajectories are depicted over the ${{\widetilde{\Omega }}}$–$\varepsilon$–${\widetilde{\sigma }}$ surface. Notice that for symmetric dissipation, ${{\widetilde{\Sigma }}}_c=1/2$, the basin of attraction is less constraint and accepts larger perturbations.

**Figure 3:**
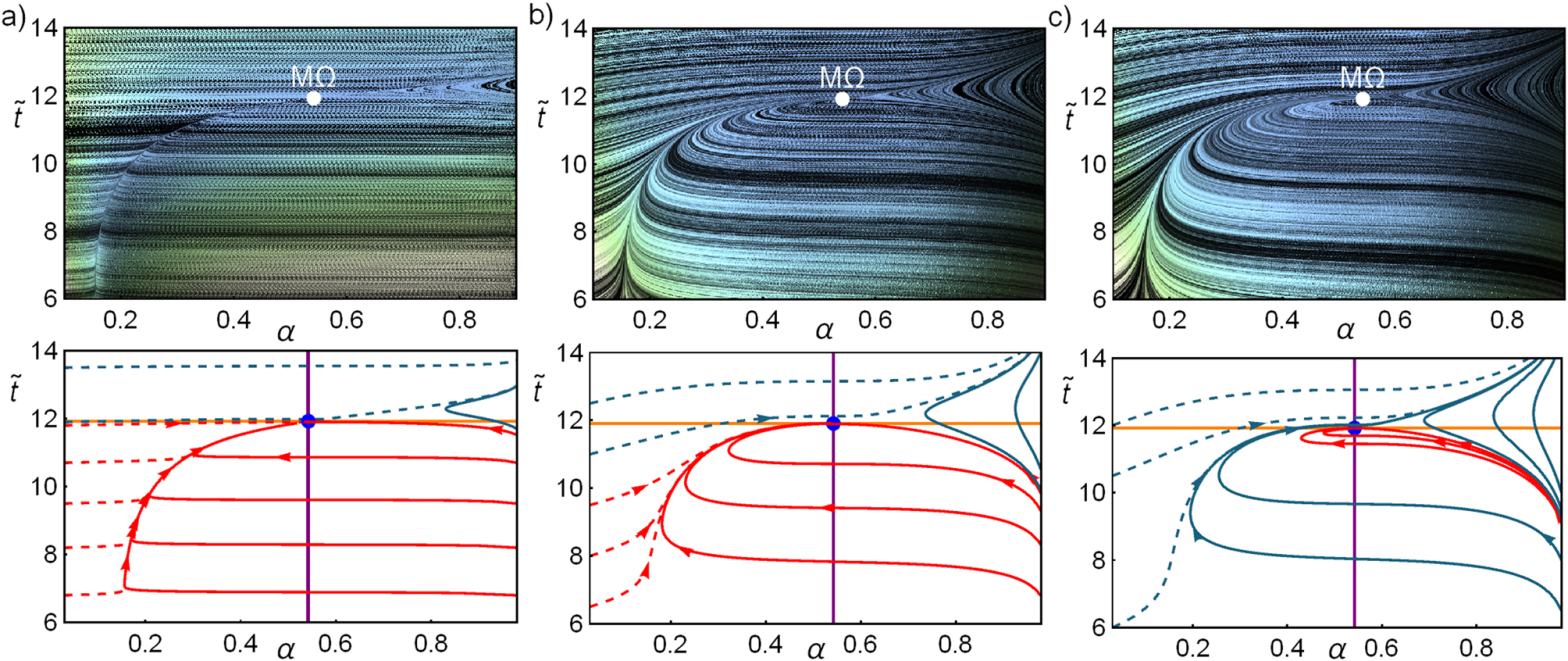
Dynamics given by solving Eqs. (9) and (10) with given initial conditions. In all cases $C=2(2-\tau )/(\tau (1-\tau ))$ and three cases for *D* are depicted: in column (**a**) $D=1$, in (**b**) $D=C$, in (**c**) $D=3/2\,C$; with $\tau =3/5$ and ${{\widetilde{\Sigma }}}_c=1/2$. In the first row, the line integral convolution plot, simulating streamlines of fixed arc length over a set of random conditions. For the second raw, continuous (dashed) lines start at $\alpha \rightarrow 1$ ($\alpha \rightarrow 0$). Red curves are located inside the basin of stability. The endoreversible limit is denoted by a vertical (purple) line and the irreversible limit by a horizontal (yellow) line.

Figure 3 shows the influence of the relation between the magnitudes of *C* and *D* in the basin of attraction. Trajectories in the phase space after a perturbation for three cases: $D=\{1,\,C,\,3/2C\}$ are presented. In the first row of Fig. 3 the line integral convolution plot of Eqs. (9) and (10) is depicted. In the second row of Fig. 3 some representative computed trajectories are shown with initial conditions corresponding to values of $\alpha \rightarrow \{0,1\}$.

**Figure 4:**
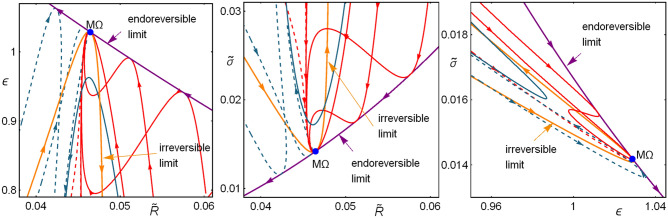
Dynamic evolution of ${{\widetilde{R}}}$, $\varepsilon$ and ${{\widetilde{\sigma }}}$ from the solution of Eqs. (9) and (10) with given initial conditions for the case $C=D=2(2-\tau )/(\tau (1-\tau ))$; with $\tau =3/5$ and $\widetilde{\Sigma }_c=1/2$. Similar behaviors are obtained for other values of *C*, *D*, $\tau$ and ${{\widetilde{\Sigma }}}_c$. Continuous lines start at $\alpha \rightarrow 1$, dashed lines at $\alpha \rightarrow 0$. Red curves correspond to those located inside the basin of stability; purple curve to the endoreversible limit (arrows indicate the direction of increasing ${{\widetilde{t}}}$) and the yellow curve to the irreversible limit (arrows indicate the direction of increasing $\alpha$).

As can be seen in Fig. 4 for the representative case $D=C$ (for $C\ne D$ the results are similar) the endoreversible and irreversible limits have meaningful information regarding the performance of the refrigerator. The COP, cooling power, and entropy generation behaviors show that the trajectories tend to approach the endoreversible limit, which represents the better energetic compromise. After arriving to the endoreversible limit, the trajectories orbit around the stable state, however, those inside the stability basin display the smallest drawback in both $\varepsilon$–${{\widetilde{\sigma }}}$ and those outside have the largest decay in the energetic efficiency and increment on entropy.

A characteristic of trajectories inside the basin of attraction is that the approaching to the endoreversible limit occurs with ${{\widetilde{t}}}<{{\widetilde{t}}}^{M\Omega }$; then, in the arrival to the stable state their orbits are bounded by the irreversible limit (yellow curve) which is tangent to the basin of attraction in the steady state. Similar features were reported in the case of HE’s. From this analysis it can be said that the endoreversible limit represents an attractor involved in an energetic self-improvement of the system and the irreversible limit bounds the basin of attraction. Both behaviors are relevant in the stability and thermodynamic improvement for both HE’s and RE’s.

### Consecutive random perturbations

It is interesting to analyze the case where there is a lack of control in the optimization variables. Fluctuations on these time-variables will lead to variations on the heat fluxes and thus fluctuations around the steady state are expected. Since the internal dynamical nature of the system is already accounted through the macroscopic description given by the low-dissipation model, the source of these fluctuations for the problem at hand comes from outside the system.

For the analysis of these fluctuations the system at M$\Omega$ conditions will undergo consecutive random perturbations along one cycle. To this purpose a cycle time will be divided by *N* equal sub-intervals of length $\Delta t$. The final state after one cycle is computed by solving the stochastic differential equation based on the proposed dynamic Eqs. (9) and (10), using a normally-distributed random variable as an additive white noise. Here, two independent stochastic variables $\xi _1$ and $\xi _2$ in the $\alpha$–${{\widetilde{t}}}$ directions follow a 2-dimensional Gaussian distribution:

14 $$\begin{aligned} f_{\xi }({{\widetilde{t}}}_c,\widetilde{t}_h)=\frac{\gamma }{2\;\pi \;\alpha ^{M\Omega }\;\widetilde{t}^{M\Omega }}\;e^{-\gamma \left[ \left( \frac{\alpha }{\alpha ^{M\Omega }}\right) ^2 +\left( \frac{\widetilde{t}}{{{\widetilde{t}}}^{M\Omega }}\right) ^2\right] }, \end{aligned}$$

A representative case, $\gamma =2/\Delta t^2\approx 2\times 10^{-8}$ is considered; thus, the standard deviations are $\sigma _{\widetilde{t}}={{\widetilde{t}}}^{M\Omega }\Delta t/\sqrt{2}\approx 8.4\times 10^{-4}{{\widetilde{t}}}^{M\Omega }$ and $\sigma _{\alpha }=\alpha ^{M\Omega }\Delta t/\sqrt{2}\approx 8.4\times 10^{-4}\alpha ^{M\Omega }$. By using the Euler–Maruyama method^58^ the points in the phase space are calculated iterating

15 $$\begin{aligned} \widetilde{t}_{i+1}= & {} C\left( \dot{{\widetilde{Q}}}_c\big (\alpha ^{M\Omega }, {\widetilde{t}}^{M\Omega }\big )-\dot{{\widetilde{Q}}}_c\big (\alpha _i,{\widetilde{t}}_i\big )\right) \Delta t+\xi _1\sqrt{\Delta t}+{{\widetilde{t}}}_{i}, \end{aligned}$$

16 $$\begin{aligned} \alpha _{i+1}= & {} D\left( \;{\widetilde{\Omega }}(\alpha ^{M\Omega }, {\widetilde{t}}^{M\Omega })\;-\;{\widetilde{\Omega }}(\alpha _i,{\widetilde{t}}_i)\right) \Delta t+\xi _2\sqrt{\Delta t}+\alpha _{i}, \end{aligned}$$

where $(\alpha _{1},\widetilde{t}_{1})=(\alpha ^{M\Omega },{\widetilde{t}}^{M\Omega })$; $\Delta t$ is $10^{-4}$th of a cycle period ${{\widetilde{t}}}^{M\Omega }$, the response case $C=D$ is studied. The energetic functions and the position in the phase space are averaged in each cycle. Then, it will be assumed that after each cycle-time the system evolves or it is driven to its time-periodic steady state, without any information from past cycles. This procedure is repeated for $10^5$ trajectories. We have checked that this number of trajectories gives a good convergent statistics according to the relative entropy measured by Kullback–Leibler divergence^56^ (see “A.1”), meaning that adding more trajectories do not offer further information and that the statistical description is conclusive.

**Figure 5:**
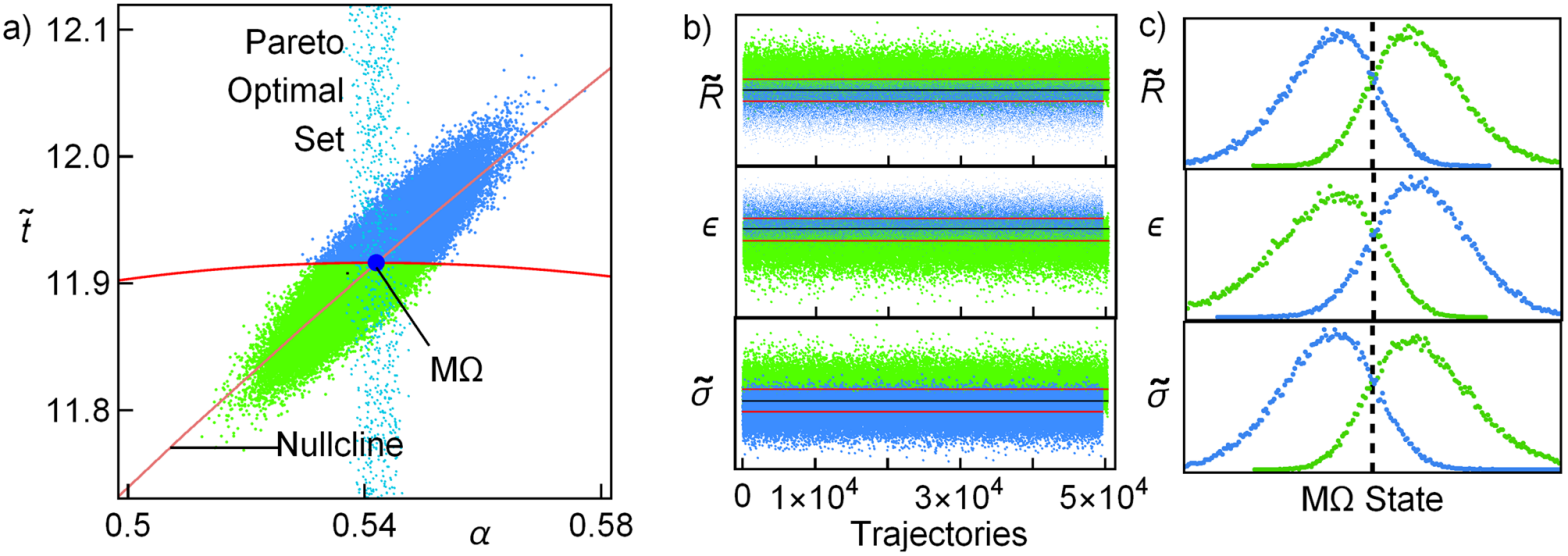
In (**a**) the final states after $10^5$ cycles, each with $10^4$ perturbations. In blue the points corresponding to states outside the stable region and in green points inside the stable region. There are slightly more points inside the stability region. The perturbations in this case allow for larger perturbations: $\{\sigma _{\alpha },\,\sigma _{\widetilde{t}}\}=\{\alpha ^{M\Omega }\,\Delta t/\sqrt{2},\widetilde{t}^{M\Omega }\,\Delta t/\sqrt{2}\}$; the relaxation time considered is $t_1={{\widetilde{t}}}^{M\Omega }/2$ and $C=D$, $\tau =2/3$, ${{\widetilde{\Sigma }}}_c=1/2$. In (**b**) the mean values of the energetic properties obtained for each cycle. The mean values for all trajectories are depicted as horizontal lines. In (**c**) the probability distribution function for the same energetic functions. For computing this, the size of the bins, or intervals, is such that the range of values of every function is distributed in $\sqrt{10^5}$ (square root of the number of trajectories) equal intervals.

Figure 5a shows the final states in the phase space after each cycle: in green the points that ended inside the basin of attraction and in blue those that ended outside. For this kind of dynamics there exists a nullcline given by the curve ${{\widetilde{t}}}(\alpha )$ for which ${{\dot{\alpha }}}=0$ (see the returning point in the $\alpha$ direction in trajectories evolving from large values of $\alpha$ in Fig. 5). The consequences of this is that $\alpha$ evolves slowly around this curve. Note in Fig. 5a the relevant influence of the nullcline on the final point locations, which are agglomerated around it. The number of points inside the attraction basin is slightly larger than outside. After each cycle is completed, the arithmetic mean of each thermodynamic function is calculated. Points inside the basin of attraction tend to have shorter contact times with the cold reservoir and also shorter total operation times, while the opposite occurs for the points outside the basin of attraction. The Pareto front is depicted to compare the locus of the final states with the best thermodynamic compromise. The mean values obtained for all trajectories are depicted in Fig. 5b as horizontal lines. In Fig. 5c the corresponding probability distribution functions for ${{\widetilde{\Omega }}}$, $\varepsilon$, and ${{\widetilde{\sigma }}}$ show the distinctive behavior for points inside and outside the stability region.

**Figure 6:**
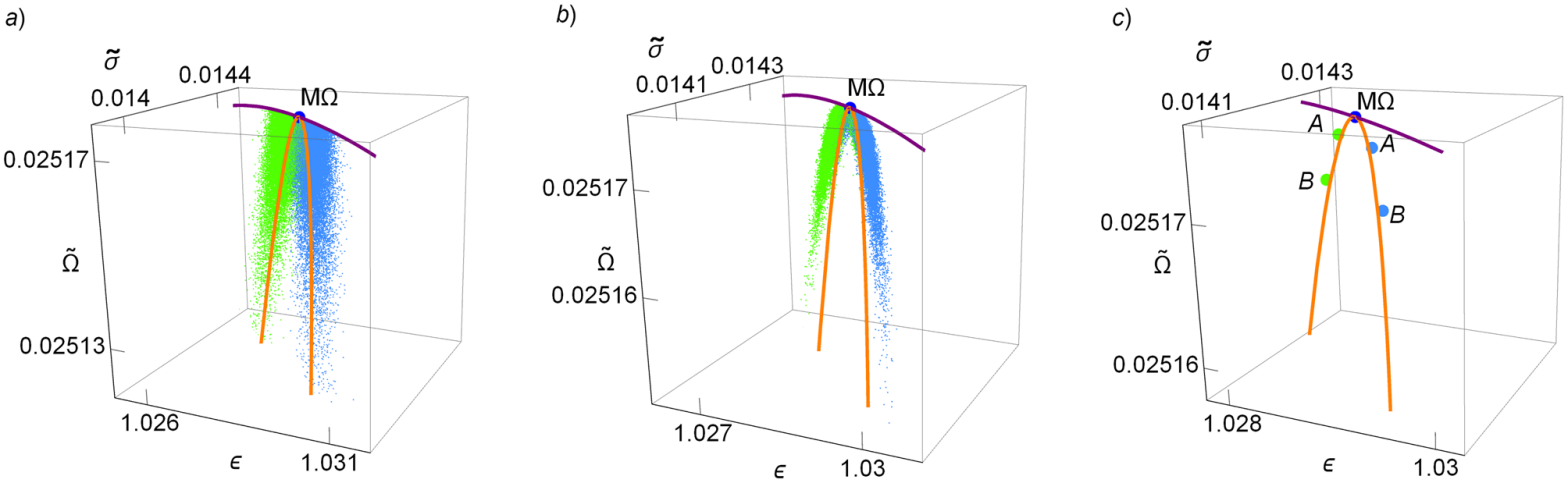
In (**a**) ${{\widetilde{\Omega }}}$, $\varepsilon$ and ${{\widetilde{\sigma }}}$ are evaluated in the final states of each $10^5$ trajectories. The averages of these points are labeled as “B” in subfigure (**c**). In (**b**) the averaged values of ${{\widetilde{\Omega }}}$, $\varepsilon$ and ${{\widetilde{\sigma }}}$ along each trajectory (cycle) are displayed and the average of these states correspond to the points label with “A” in subfigure (**c**). In (**c**) the mentioned averaged thermodynamic functions are presented to stress their closeness to the irreversible limit given by the orange loop-like curve and the upper bound fixed by the endoreversible condition (purple curve).

In Fig. 6a, the final states after each cycle are displayed in the ${{\widetilde{\Omega }}}$–*$\varepsilon$–${{\widetilde{\sigma }}}$ space. The endoreversible limit establishes an upper bound for all these trajectories and below the energetic configurations are located around the irreversible limit. In Fig. 6b the averaged states over each stochastic trajectory/cycle are presented. Finally, in Fig. 6c the label “A” indicates the averaged energetic states for the mean values, and the label “B” the average of the final states, both points show that the performance of many cycles is very closed to that of the irreversible limit.

This part of the analysis points out to a key role of time constraints in the obtaining of endoreversible and irreversible behaviors and their implications on the overall thermodynamic performance when the operation regime is stochastically perturbed. Many works have been reported on the endoreversible limit and its validity range. The stochastic results here obtained from induced perturbations on a steady state give new insights about the status and true nature of this endoreversible limit: it behaves as an attractor of the overall dynamics and describes the best compromise between the most relevant energetic functions, while the irreversible limit distinguishes trajectories inside or outside the stability basin and under perturbations it describes the statistical irreversible behavior induced over the system. These results are reproduced for different random variables distributions and *C*, *D* combinations.

## Relaxation velocities and self-optimization

A second pair of time-variables can be used to study the optimization and stability of the LD RE. The optimization analysis is analogous to that of the previous Section, but the dynamics involved in the stability is different and as it will be shown below, the operation regime is described through a global stable state. This will provide a complementary vision of the linking between optimization and stability.

By introducing the dimensionless variables $\widetilde{t}_c=t_c\,\Delta S/\Sigma _h$, $\widetilde{t}_h=t_h\,\Delta S/\Sigma _h$, the input and output heats are ${\widetilde{Q}}_c=Q_c/(T_h\Delta S)$, ${\widetilde{Q}}_h=Q_h/(T_h\Delta S)$, respectively, and $\Sigma =\Sigma _c/\Sigma _h$ (taking under consideration the system size). The heat exchanges per cycle can be written as

17 $$\begin{aligned} {\widetilde{Q}}_c=\tau -\frac{\tau \,\Sigma }{\widetilde{t}_c}; {\widetilde{Q}}_h=-1-\frac{1}{\widetilde{t}_h}, \end{aligned}$$

in this way, the total entropy change of the thermodynamic universe per cycle is $\widetilde{\Delta S}_{tot}=({\widetilde{t}_h}^{-1}+\Sigma \;{{{\widetilde{t}}}_c}^{-1}$) and the entropy production ${{\widetilde{\sigma }}}=\widetilde{\Delta S}_{tot}/(\widetilde{t}_c+{{\widetilde{t}}}_h)$. The information related to the internal dynamics is accounted by $\Sigma$. Together with $\tau$, it will remain as a fixed parameter. Due to the normalizing definitions the fluxes ${{\widetilde{R}}}\equiv {{\widetilde{Q}}}_c/({{\widetilde{t}}}_c+\widetilde{t}_h)$, ${{\widetilde{P}}}_{in}$, ${{\widetilde{\sigma }}}$ and ${{\widetilde{\Omega }}}$ differ from the functions appearing in the previous section by a factor $(1+\Sigma )$. The optimization of $\Omega$ is achieved through the partial contact times $\widetilde{t}_c$ and ${{\widetilde{t}}}_h$:

18 $$\begin{aligned} \widetilde{t}_h^{M\Omega }=\frac{2}{\left( 1-\tau \right) } \left( 1+\sqrt{\Sigma \left( 2-\tau \right) }\right) ;\quad \widetilde{t}_c^{M\Omega }=\sqrt{\Sigma \left( 2-\tau \right) } \,\widetilde{t}_h^{M\Omega }. \end{aligned}$$

The same upper and lower bounds for $\varepsilon ^{M\Omega }$ appearing in Eqs. (2) and (3) are obtained for $\Sigma \rightarrow 0$ and $\Sigma \rightarrow \infty$, respectively.

### The best energetic compromise

**Figure 7:**
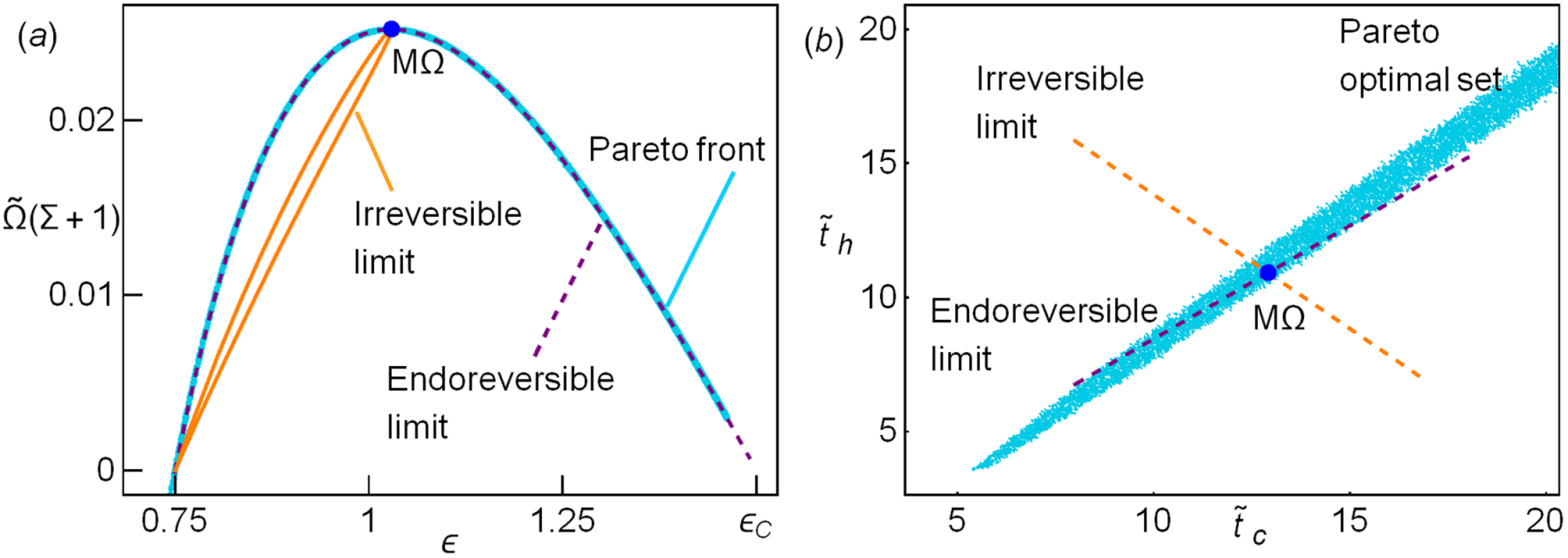
(**a**) The Pareto front (cyan points) matches the endoreversible limit (parabolic dashed line). The term $1+\Sigma$ is the scale factor between the two normalized $\Omega$s (compare with Fig. 1a). For completeness, the irreversible limit is also depicted (loop-like curve). (**b**) The Pareto optimal set is shown (cyan points). The irreversible limit and irreversible limits are shown. The representative values $\tau =3/5$ and $\Sigma =1$ have been considered.

The same method to obtain the Pareto front (see in “The best energetic compromise” section) is used for this set of variables. The resulting Pareto front is depicted in Fig. 7a and coincides with that appearing in Fig. 1a with a scale factor $(1+\Sigma )$. The Pareto optimal set is shown in Fig. 7b. The endoreversible and irreversible behaviors stemming from the time constraints are plotted in this pair of variables, see Fig. 7b.

As mentioned before, the two pairs of time variables discussed in the previous section and in this one are equivalent, the Pareto front, as well as the Pareto optimal set can be mapped from one system into the other and the exact results are obtained. Now, the dynamic equations for the second set of variables will be introduced, but one must notice that in the previous section the quantities that is tried to be fixed are energy fluxes, which is specially relevant in irreversible stationary processes. In this case, on the other hand, the stability is described in terms of a fixed energy (see below). For that reason, both dynamics are not equivalent.

### Stability dynamics

Now, time variables are associated to heat exchanges between the system and the surroundings. It is then plausible to model the external perturbations on the operation regime as variations on the input and output heat, affecting engine operation time. For this analysis $\tau$ and $\Sigma$, which are intrinsic properties of the system, will remain fixed. Each heat only depends on the associated partial contact time, i. e., ${{\widetilde{Q}}}_c=\widetilde{Q}_c({{\widetilde{t}}}_c)$ and ${{\widetilde{Q}}}_h={{\widetilde{Q}}}_h(\widetilde{t}_h)$ [see Eq. (17)], then, variations on the contact times can be effectively linked to variations on the corresponding input/output heats. The matrix formulation given in the previous section can now be addressed as a first-order one-dimensional uncoupled system^57^. The autonomous equations for the dynamics now are

19 $$\begin{aligned} \frac{d}{dt}\big (\widetilde{t}_c-{{\widetilde{t}}}_c^{M\Omega }\big )\propto -\big (\widetilde{t}_c-{{\widetilde{t}}}_c^{M\Omega }\big );\quad \frac{d}{dt}\big (\widetilde{t}_h-{{\widetilde{t}}}_h^{M\Omega }\big )\propto -\big (\widetilde{t}_h-{{\widetilde{t}}}_h^{M\Omega }\big ). \end{aligned}$$

Additionally, a first order expansion of ${{\widetilde{Q}}}_c$ and ${{\widetilde{Q}}}_h$ around the steady state gives

20 $$\begin{aligned}&{{\widetilde{Q}}}_c\left( {{\widetilde{t}}}_c\right) -\widetilde{Q}_c\left( {{\widetilde{t}}}_c^{M\Omega }\right) =\left. \frac{d\widetilde{Q}_c\left( {{\widetilde{t}}}_c\right) }{d\widetilde{t}_c}\right| _{M\Omega }\left( {{\widetilde{t}}}_c-\widetilde{t}_c^{M\Omega }\right) , \end{aligned}$$

21 $$\begin{aligned}&{{\widetilde{Q}}}_h\left( {{\widetilde{t}}}_h\right) -\widetilde{Q}_h\left( {{\widetilde{t}}}_h^{M\Omega }\right) =\left. \frac{d\widetilde{Q}_h\left( {{\widetilde{t}}}_h\right) }{d\widetilde{t}_h}\right| _{M\Omega }\left( {{\widetilde{t}}}_h-\widetilde{t}_h^{M\Omega }\right) . \end{aligned}$$

By combining Eqs. (19)–(21), it is possible to provide a dynamics linking the contact times with variations in the input/output heats as follows

22 $$\begin{aligned} \frac{d\widetilde{t}_c}{dt}=A\left( {\widetilde{Q}}_c(\widetilde{t}_c^{*}) -{\widetilde{Q}}_c(\widetilde{t}_c)\right) ;\quad \frac{d\widetilde{t}_h}{dt}=B\left( {\widetilde{Q}}_h(\widetilde{t}_h^{*}) -{\widetilde{Q}}_h(\widetilde{t}_h)\right) , \end{aligned}$$

where *A* and *B* are positive constants, giving the restitution strength. Their values may depend on multiple characteristics, but usually the system size is the most important of them: the larger the system the smaller the values of *A* and *B*. From a dynamical perspective, the inverse values of *A* and *B* set a characteristic time scale. In the forthcoming analyses, all the results are referred to this time scale.

In a linear approximation the local stability of this steady state is determined by the eigenvalues, $\lambda _1$ and $\lambda _2$, and eigenvectors of the Jacobian matrix:

23 $$\begin{aligned} {\mathscr{J}}=- \left[ \begin{array}{cc} A\frac{\tau \Sigma }{{t_c^{M\Omega }}^2} &{}\quad 0 \\ 0 &{}\quad B\frac{1}{{t_h^{M\Omega }}^2} \end{array} \right] =\left[ \begin{array}{cc} \lambda _1 &{}\quad 0 \\ 0 &{}\quad \lambda _2 \end{array} \right] . \end{aligned}$$

and since both $\lambda _1$ and $\lambda _2$ are real and negative, the operation regime steady state is stable. Relaxation times are defined as $t_1\equiv -1/\lambda _1$ and $t_2\equiv -1/\lambda _2$, and by using Eqs. (18) and (23) they are given by

24 $$\begin{aligned} t_1=\frac{4\left( 1+\sqrt{\Sigma \left( 2-\tau \right) } \right) ^2}{A\left( 1-\tau \right) ^2}\left( \frac{2-\tau }{\tau }\right) ;\quad t_2=\frac{4\left( 1+\sqrt{\Sigma \left( 2-\tau \right) }\right) ^2}{B\left( 1-\tau \right) ^2}. \end{aligned}$$

From the above equations it is easy to check that relaxation is dominated by $t_1$ (linked to ${{\widetilde{t}}}_c$). This is more noticeable for small values of $\tau$ and large values of $\Sigma$, that is, when the dissipation coefficient at the cold reservoir is larger. By defining the total operation time $\widetilde{t}^{M\Omega }_{tot}\equiv \widetilde{t}_h^{M\Omega }+\widetilde{t}_c^{M\Omega }$, that from Eq. (18) is given by

25 $$\begin{aligned} \widetilde{t}^{M\Omega }_{tot}=\frac{2}{1-\tau }\left( 1+\sqrt{\Sigma \left( 2-\tau \right) } \right) ^2, \end{aligned}$$

the operation time can be compared with the total relaxation time, defined by $t_{relax}\equiv t_1+t_2$,

26 $$\begin{aligned} t_{relax}=\frac{4\left( 1+\sqrt{\Sigma \left( 2-\tau \right) }\right) ^2}{\left( 1-\tau \right) ^2}\left( \frac{2-\tau }{A\;\tau }+\frac{1}{B}\right) =\frac{2}{1-\tau }\left( \frac{2-\tau }{A\;\tau }+\frac{1}{B}\right) \,\widetilde{t}^{M\Omega }_{tot}. \end{aligned}$$

Since it is desirable that the system returns to the steady state within a cycle period $t_{relax}\le {\widetilde{t}}^{M\Omega }_{tot}$, *A* and *B* should fulfill the following condition

27 $$\begin{aligned} \frac{2-\tau }{A\;\tau }+\frac{1}{B}\le \frac{1-\tau }{2}. \end{aligned}$$

**Figure 8:**
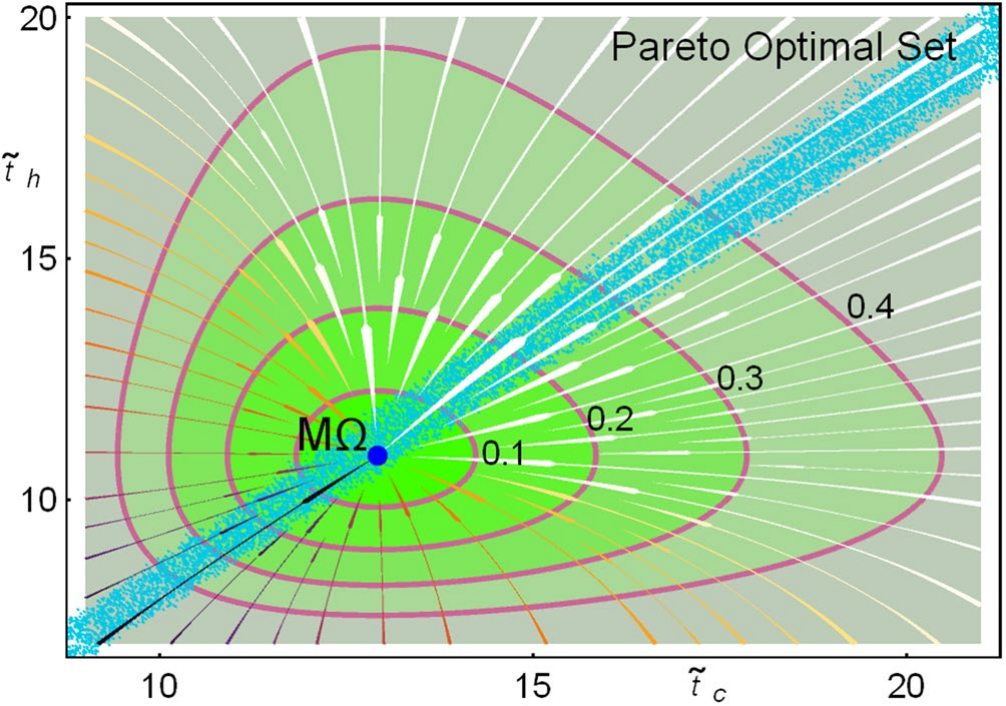
Stream plot of the dynamics given by Eq. (22) for the case where $t_1=t_2$. Level curves of constant velocities ($\sqrt{(d{{\widetilde{t}}}_c/dt)^2+(d\widetilde{t}_h/dt)^2}=const$) are displayed to show not only the patterns over time, but also how rapidly the velocity changes as the system heads towards the steady state. In both cases the fastest velocity transitions occur in the region where ${{\widetilde{t}}}_{c,h}<\widetilde{t}^{M\Omega }_{c,h}$.

Beyond the linear approximation, the system given by Eq. (22) can be solved numerically. Figure 8 shows the stream plot of the dynamics, which exhibits a stable point. Level curves for the dynamical velocity $v_{dyn}\equiv \sqrt{(d\widetilde{t}_c/dt)^2+(d{{\widetilde{t}}}_h/dt)^2}=const$ are depicted. Notice that the velocity of relaxation is different in each quadrant. In this case the constraint $t_1=t_2$ is used. Shorter relaxations are achieved for ${{\widetilde{t}}}_c<{{\widetilde{t}}}_c^{M\Omega }$ and ${{\widetilde{t}}}_h<{{\widetilde{t}}}_h^{M\Omega }$, for other constraints, such as $A=B$ this qualitative behavior holds, but the asymmetry between relaxation times will affect these contours. On the other hand, the Pareto optimal set, which do not depend on the restitution dynamics is depicted as a reference. The points in the Pareto optimal set with larger contact times (slower operation time) are closer to the minimum entropy generation state, meanwhile those with smaller contact times (faster operation time) are closer to the maximum *R* regime. One remarkable feature is that it appears a region where transition between velocity contours are more separated, meaning that restitution takes longer compared with changes in operation time. On the other hand, for another region transitions are faster. The largest restitution times are exhibited when only one partial time is perturbed. This will have consequences in the statistics of consecutive perturbations, as will be shown in next subsection.

**Figure 9:**
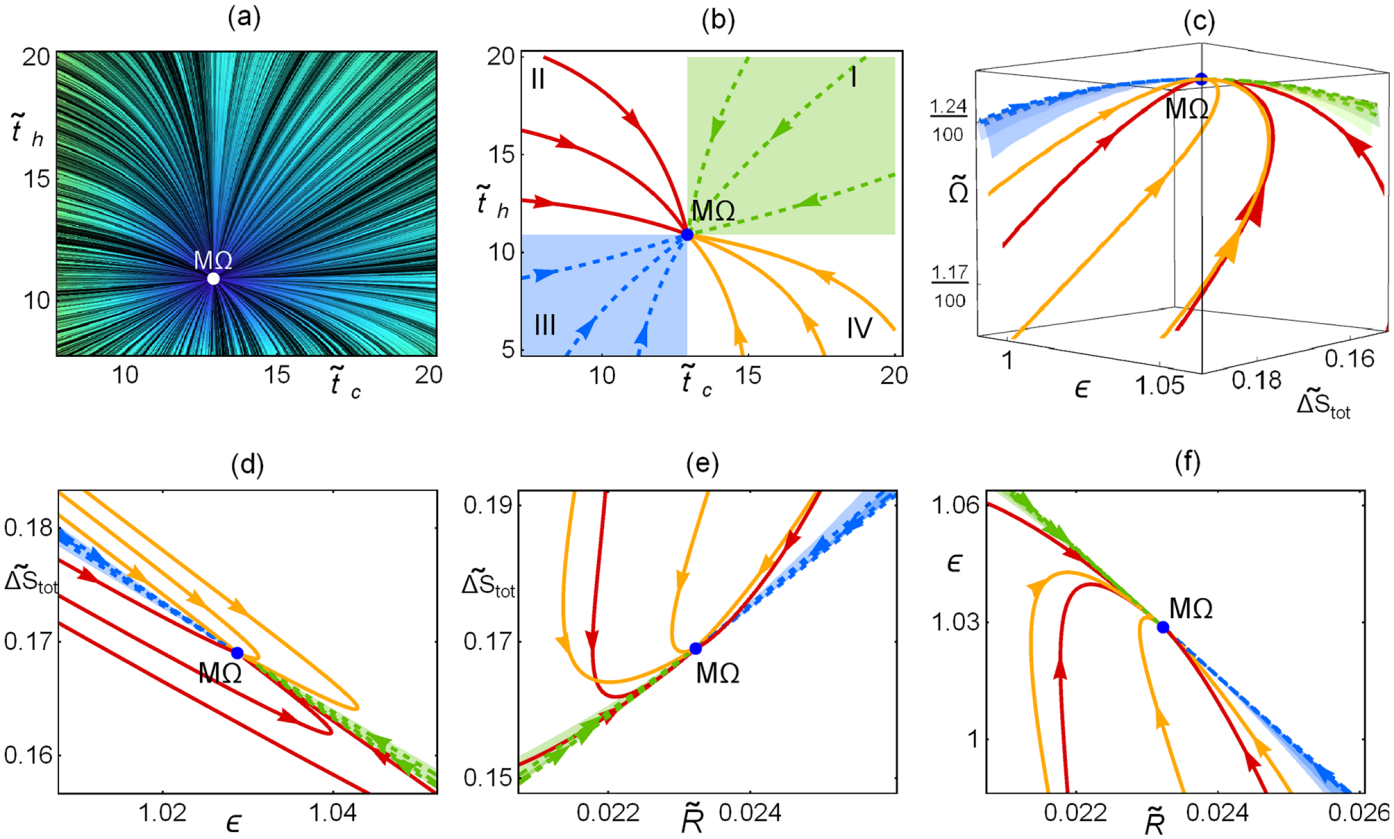
(**a**) Line integral convolution plot of the dynamic equations (22), simulating streamlines over random conditions. (**b**) Trajectories towards relaxation in the phase space. (**c**) Trajectories in the $\varepsilon$–*${{\widetilde{\Omega }}}$–${{\widetilde{\sigma }}}$ surface. (**d**–**f**) 2D-plots for the entropy generation, cooling power and COP. In all cases $\Sigma =1$, $\tau =3/5$ and *A* and *B* are chosen to fulfill that $t_1=t_2$ and $t_{relax}=\widetilde{t}_{tot}^{M\Omega }$.

In Fig. 9a the line integral convolution plot of the dynamic equations (22) over a random distribution of initial conditions is depicted, simulating streamlines of fixed arc length; darker shaded regions indicates smaller velocities.

Figure 9b shows some representative trajectories with initial conditions at the border of the depicted region (initial state after a perturbation). Trajectories in each quadrant, labeled as *I*–*IV* are represented with different colors to emphasize differences in the energetic planes plotted in Fig. 9c–f. Trajectories in each quadrant evolve in a slightly different way, but this fact yields noticeable energetic repercussions. Of special relevance are regions I and III (colored in green and blue, respectively).

Figure 9c shows the trajectories in the $\widetilde{\Delta S}_{tot}$–$\varepsilon$–$\Omega$ surface. It is observed that trajectories in quadrants II and IV present large variations in the COP and entropy change before arriving to the stable state. This last feature could be considered as a non-desirable behavior. On the other hand, a kind of “benefit” in quadrants I and III is obtained since trajectories in these regions produce the best compromise between a given cooling power and the COP (larger values of $\varepsilon$ for given ${{\widetilde{R}}}$) and the entropy change (the less entropy changes for a given ${{\widetilde{R}}}$) at the same time. These features are detailed in the 2D-plots of Fig. 9d–f showing that the trajectories in quadrants I (green) and III (blue) evolve directly towards the stable point in a very narrow region, meanwhile trajectories in quadrants II (red) and IV (yellow) present sudden changes of direction before arriving to the steady state.

The consequences of the dynamics on the system energetic properties could imply the use of a disadvantage, such as limited control, as a self-optimization mechanism throughout a biased control focused mostly in perturbations towards quadrants II and IV, to favor perturbations with both ${{\widetilde{t}}}_c>{{\widetilde{t}}}_c^{M\Omega }$ and ${{\widetilde{t}}}_h>{{\widetilde{t}}}_h^{M\Omega }$ or $\widetilde{t}_c<{{\widetilde{t}}}_c^{M\Omega }$ and ${{\widetilde{t}}}_h<\widetilde{t}_h^{M\Omega }$.

One could be interested not only in one perturbation but a series of them affecting the performance of one cycle. The influence of continuous random perturbations over a cycle will be addressed below in order to get more insights about this issue.

### Consecutive random perturbations

To analyze the effect of the dynamics in random perturbations a cycle time will be divided by *N* equal sub-intervals of length $\Delta t$. The final state after continuous perturbations along one cycle is computed by solving the stochastic differential equation based on the proposed dynamic equations (22), using a normally-distributed random variable as an additive white noise. Here, the two independent stochastic variables $\xi _1$ and $\xi _2$ in the ${{\widetilde{t}}}_c$ and ${{\widetilde{t}}}_h$ directions follow a 2-dimensional Gaussian distribution. By using the Euler-Maruyama method^58^ phase space points are calculated iterating for the *N* steps

28 $$\begin{aligned} {{\widetilde{t}}}_{c_{i+1}}= & {} A\left( {\widetilde{Q}}_c\left( \widetilde{t}_c^{M\Omega }, \widetilde{t}_h^{M\Omega }\right) -{\widetilde{Q}}_c\left( \widetilde{t}_{c_{i}}, \widetilde{t}_{h_{i}}\right) \right) \Delta t+\xi _1\sqrt{\Delta t} +{{\widetilde{t}}}_{c_{i}}, \end{aligned}$$

29 $$\begin{aligned} {{\widetilde{t}}}_{h_{i+1}}= & {} B\left( {\widetilde{Q}}_h\left( \widetilde{t}_c^{M\Omega }, \widetilde{t}_h^{M\Omega }\right) -{\widetilde{Q}}_h\left( \widetilde{t}_{c_{i}}, \widetilde{t}_{h_{i}}\right) \right) \Delta t+\xi _2\sqrt{\Delta t}+{{\widetilde{t}}}_{h_{i}}. \end{aligned}$$

**Figure 10:**
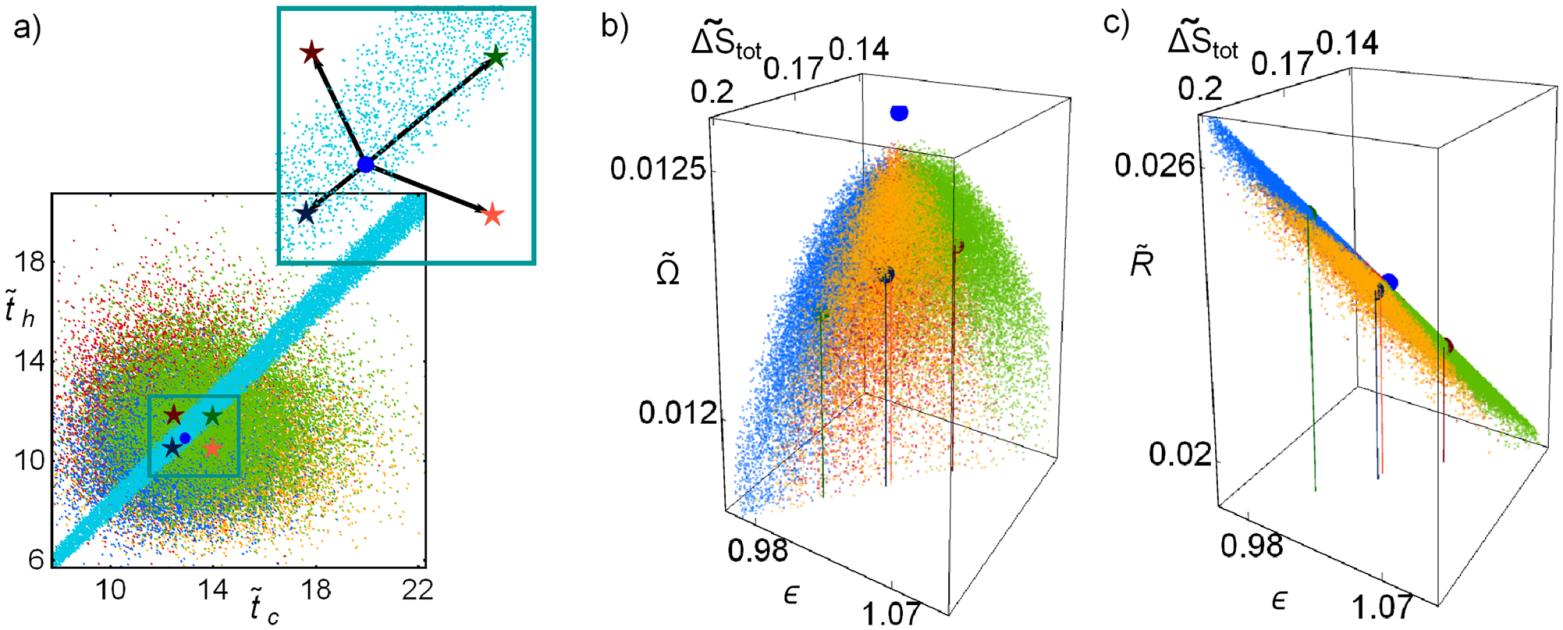
(**a**) Ending points of $5\times 10^4$ trajectories. In the inset box a close caption of the geometric centers of all the trajectories in each quadrant is displayed. Starts denote the geometric centers of the points in each quadrant according to Fig. 9b; (**b**) Averaged values of $\Delta S$, $\varepsilon$ and ${{\widetilde{\Omega }}}$, and (**c**) averaged values of $\Delta S$, $\varepsilon$ and ${{\widetilde{R}}}$. The representative values $\tau =3/5$ and $\Sigma =1$ are used, but for other values the behavior is similar.

The stochastic variables $\{\xi _1,\xi _2\}$ follow the distribution

30 $$\begin{aligned} f_{\xi }({{\widetilde{t}}}_c,\widetilde{t}_h)=\frac{\gamma }{2\;\pi \;{{\widetilde{t}}}_c^{M\Omega }\;\widetilde{t}_h^{M\Omega }}\;e^{-\frac{\gamma }{2}\left[ \left( \frac{\widetilde{t}_c}{{{\widetilde{t}}}_c^{M\Omega }}\right) ^2+\left( \frac{\widetilde{t}_h}{{{\widetilde{t}}}_h^{M\Omega }}\right) ^2\right] }, \end{aligned}$$

where $\gamma =100$, the standard deviation $\sigma _{\widetilde{t}_{c,h}}$ is ${{\widetilde{t}}}_{c,h}^{M\Omega }/10$. The initial state is $({{\widetilde{t}}}_{c_{1}},\widetilde{t}_{h_{1}})=(\widetilde{t}_c^{M\Omega },\widetilde{t}_h^{M\Omega })$; $\Delta t$ is $t^{M\Omega }_{tot}/10^4$ (25), so that after $10^4$ steps one cycle time is covered. The constants $A=4\times 10(2-\tau )/(\tau (1-\tau ))$ and $B=4\times 10/(1-\tau )$ are used, so $t_{relax}=t^{M\Omega }_{tot}/10$ [see Eq. (27)] along with the condition $t_1=t_2$, so the dynamics is almost the same in the ${{\widetilde{t}}}_{c,h}$ directions. After one cycle has ended, the system attains the time-periodic steady state and starts another random trajectory without any information regarding previous cycles. This is repeated for $5\times 10^4$ trajectories/cycles. The statistical convergence has been tested using the Kullback–Leibler divergence^56^ of the system energetic distributions [see equation (“A.2”)]. The results are shown in Fig. 10.

In Fig. 10a the final states for each trajectory are depicted; colors indicate the quadrant of the system average position. The geometric centers of the points in each quadrant are displayed (see stars). The Pareto optimal set is shown as well. Notice that average behavior of the system is displaced towards the region of slow relaxation, following the direction of the Pareto front at which entropy change is minimized. By looking at the number of trajectories averaged in each region a trend is found: around $30.066\%$ of the trajectories stayed in region I, $20.374\%$ in region III and $49.56\%$ almost equally distributed in regions II and IV, as it is emphasized in the close caption of Fig. 10a.

The mean values for the thermodynamic functions in each cycle are depicted in Fig. 10b–c for the energetic space involving $\widetilde{\Delta S}_{tot}$, $\varepsilon$, ${{\widetilde{\Omega }}}$ and ${{\widetilde{R}}}$. The color representation is maintained. Points in region I have the best performance involving entropy production and efficiency, meanwhile trajectories in region III (region of fast relaxations) maintain closer to the steady state. In regions II and IV the energetic functions fluctuate very close to the steady state. From the above discussion the stability dynamics favors an energetic behavior where efficiency and entropy changes are improved at the cost of decreasing cooling power.

## Concluding remarks and perspectives

The local stability of the Maximum Omega regime has been analyzed for the low dissipation refrigerator under two complementary dynamics. One with perturbations on one heat flux and the Omega function and another one with perturbations on the input/output heats. For each one an equivalent pair of time variables, which suitable describe the system and provide analogous optimizations reveal different features linking optimization with self-optimization. The restitution forces are modeled by means of dynamic equations stemming from requiring a steady state and small deviations of the input/output heat and fluxes.

On the one hand, a dynamic with a basin of attraction is obtained, along with a nullcline which outside the attraction basin, leads the system to a non-physical region. The analysis of relaxation trajectories indicates that relaxation paths evolve in such a way that the thermodynamic performance of the system is improved. The system moves directly to the endoreversible limit due to the restitution dynamics. This endoreversible limit coincides with the Pareto front, which represents the best energetic compromise between all the relevant thermodynamic functions. Thus, the restitution dynamics induces an optimization process. For the case of many perturbations along one cycle the stability dynamics constitutes an irreversible mechanism, characterizing points inside and outside the stability basin.

On the other hand, for the second dynamics (and 2nd pair of variables), a global steady state is obtained and an analysis of relaxation velocities is presented. Fast and slow relaxations are obtained and together with the Pareto front (and the endoreversible limit) have an influence in describing the case of many perturbations. When the system moves to a worse energetic state, it improves due to the restitution dynamics in a fast manner, meanwhile if the system is perturbed to a better state, it evolves slowly to the steady state. This will produce a small departure in the average behavior of the system to a more optimum state regarding entropy generation and larger efficiency (COP).

The improvement due to perturbations on the steady state is related with the phenomenon of antifragility, in which a system exhibits an improvement under stress^62^. This could be of particular interest in medicine and biological systems. The extension of the low dissipation model to chemical engines^10^ could also provide a path to test properties such as antifragility in biochemical processes.

The analysis here presented for RE’s has been reported for low dissipation heat engines as well^45, 46^, reinforcing the idea of a universal energetic characterization of heat devices as they are perturbed externally. Remarkably, the analyses of refrigeration systems are nowadays increasing its relevance since their coupling with other energy converters from different nature are being under study as an strategy for solving the problem of energy storage and heat recovery^59,60^. The study on a setup with fixed cooling power would be of interest, since in many applications the objective of refrigeration is to maintain this quantity as steady as possible.

Ideally, cyclic processes should be able to operate in a time-periodic steady state, one can make the idealization that the starting and ending points are well defined and analyze this as a unitary process. In reality, the switching between, aerobial respiration (linked to energetic trade-off functions) and anaerobial respiration (maximum power^61^), to name an example, as well as the transient states in between in the changing of regime are beyond the simplified description of the present model. But it is precisely in the connection with this unknown mechanisms, where adaptation and robustness play a major role. So far, the results presented here for the self-optimization correspond to an emergent phenomenon (theoretically) that should be tested in specific structure dependent models.

The results presented in the present paper could open the window to the joint analysis of stability and optimization of coupled energy conversion systems with different purposes (heat engines, refrigerators, and heat pumps) and with different size scales, focusing on improving the strategies of production (aiming for a sustainability) and seize on energy. Possible applications of these results might involve colloidal particles constrained in an optical trap, describing Carnot or Stirling cycles. The equivalence between low dissipation model, irreversible Curzon–Ahlborn engine and Otto and Brayton engines might also allow for applications on solar energy heat devices.

Additionally, this study could open new perspectives to analyze possible self-optimization or organization mechanism in specific systems and could be useful to understand non-revealed properties of non-equilibrium systems and their energetic bounds.

## Figures and Tables

**Figure 11:**
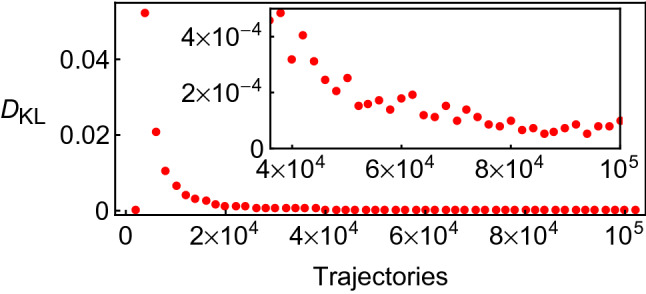
Kullback–Leibler divergence comparison at every 2000 trajectories/cycles. A good convergence is obtained for the number of trajectories analyzed in this Section.

**Figure 12:**
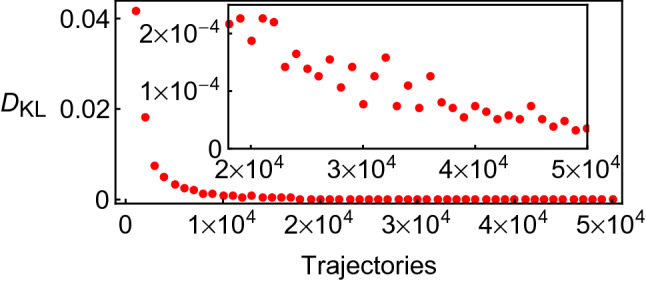
$D_{KL}$ at every $10^3$ trajectories/cycles. Convergence is considered at $5\times 10^4$ cycles.

## A Statistical convergence: Kullback–Leibler divergence

### A.1 Dynamics involving $Q_c$ and $\Omega$

The Kullback–Leibler divergence, $D_{KL}$ allows to test statistical convergence. The value of $D_{KL}$ gives a measure of how distant are two distributions. If $D_{KL}=0$, the information stemming from both distributions is the same. This is a relevant issue to demonstrate that the obtained trend is not due to the lack of additional data.

From the averaged cooling power ${{\widetilde{R}}}$ is computed of $10^5$ trajectories, the interval between the largest and smallest ${{\widetilde{R}}}$ values is divided by $\sqrt{N}$ (rounded to the upper next integer^63^) equal intervals, or bins, in this way, the same partition is used for the to compute the discrete probability distributions of the first *k*-thousand trajectories, $\rho _k$ are obtained, and the $D_{KL}$ is calculated comparing $\rho _{k-1}$ with $\rho _k$. The entropy divergence, $D_{KL,k}$ is given by

31 $$\begin{aligned} D_{KL,k}(\rho _{k-1}\Vert \rho _{k})=-\sum _{i}\rho _{k-1,i}\,log \left( \frac{\rho _{k,i}}{\rho _{k-1,i}}\right) . \end{aligned}$$

giving a measure of how much information is gained by adding more trajectories. In Fig. 11, it is is shown that from $4\times 10^4$ trajectories the statistical behavior does not vary significantly and adding trajectories will not provide much further information.

### A.2 Dynamics involving $Q_c$ and $Q_h$

Analogously, the averaged ${{\widetilde{R}}}$ values are considered for $5\times 10^5$ trajectories. The resulting $D_{KL,k}$ values are shown in Fig. 12 for $N=10^5$ trajectories, showing that from $6\times 10^4$ trajectories the statistical behavior does not vary significantly and adding more trajectories will not give much further information.
